# Diagnostic and Pathophysiological Impact of Myocardial MIBG Scintigraphy in Parkinson's Disease

**DOI:** 10.4061/2010/295346

**Published:** 2009-12-22

**Authors:** Jörg Spiegel

**Affiliations:** Department of Neurology, Saarland University, Kirrberger Straße, 66421 Homburg/Saar, Germany

## Abstract

Myocardial MIBG scintigraphy is established in the diagnosis and differential diagnosis of Parkinson's disease (PD). Numerous studies address the pathophysiological impact of myocardial MIBG scintigraphy: the myocardial MIBG uptake correlates with the clinical phenotype of PD; the background of this phenomenon is unclear. Furthermore MIBG scintigraphy enables to study the extracranial Lewy body type-degeneration. In combination with cerebral dopamine transporter imaging, MIBG scintigraphy allows to correlate cerebral and extracranial Lewy body type-degeneration in PD.

## 1. Introduction

Metaiodobenzylguanidine (MIBG) is a norepinephrine (noradrenaline) analogue which competes with norepinephrine for the same cellular transporter mechanisms of postganglionic adrenergic neurons. Like norepinephrine, MIBG is actively transported into noradrenaline granules of sympathetic nerve terminals by the noradrenaline transporter (uptake 1 mechanism; [1]). MIBG has no pharmacological activity. Due to its affinity to sympathetic nerve endings MIBG accumulates predominantly in organs with a high sympathetic activity such as the adrenal gland, the liver, the spleen, the heart, and the salivary glands [2]. For diagnostic purposes MIBG is marked by a radioactive isotope, for example, 123-iodine (^123^I). The accumulation of MIBG is visualized and measured by planar whole-body scintigraphy. In clinical practice, the MIBG scintigraphy is mainly used to detect (and sometimes to treat) neuroendocrine tumours, primarily pheochromocytomas and neuroblastomas [3–8].

## 2. MIBG Scintigraphy in the Diagnosis of Parkinson's Disease

**Figure 1:**
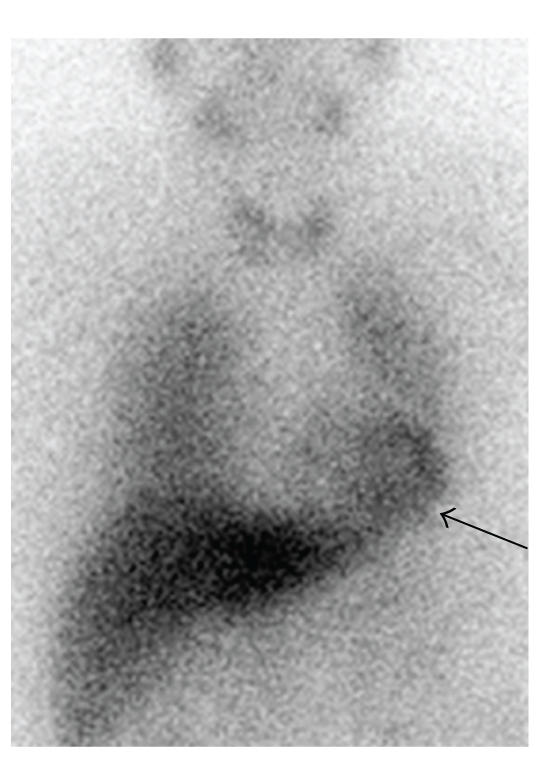
MIBG scintigraphy in a healthy volunteer. Anterior-posterior view in a 64-year-old healthy volunteer. The measurement was performed four hours after intravenous MIBG application. There is an intensive myocardial MIBG uptake (black arrow).

**Figure 2:**
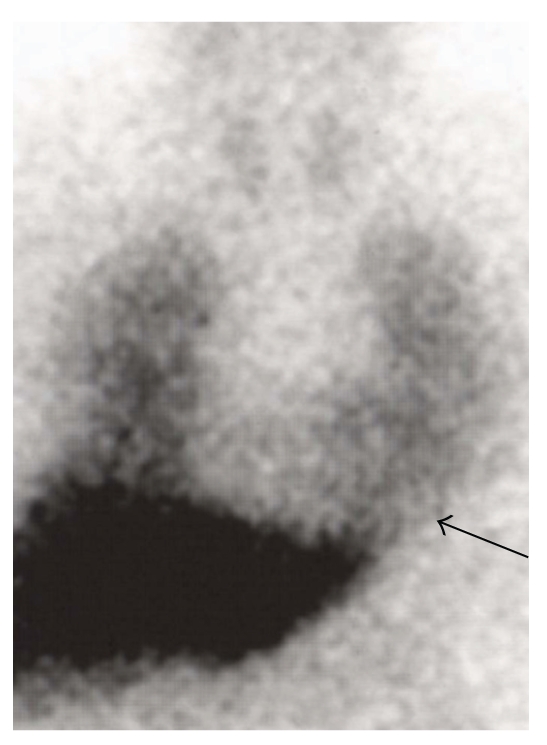
MIBG scintigraphy in tremor dominant PD. MIBG scintigraphy in a 62-year-old male patient with tremor dominant PD, Hoehn and Yahr stage I. The myocardial MIBG uptake (black arrow) is well visible but pathologically reduced.

**Figure 3:**
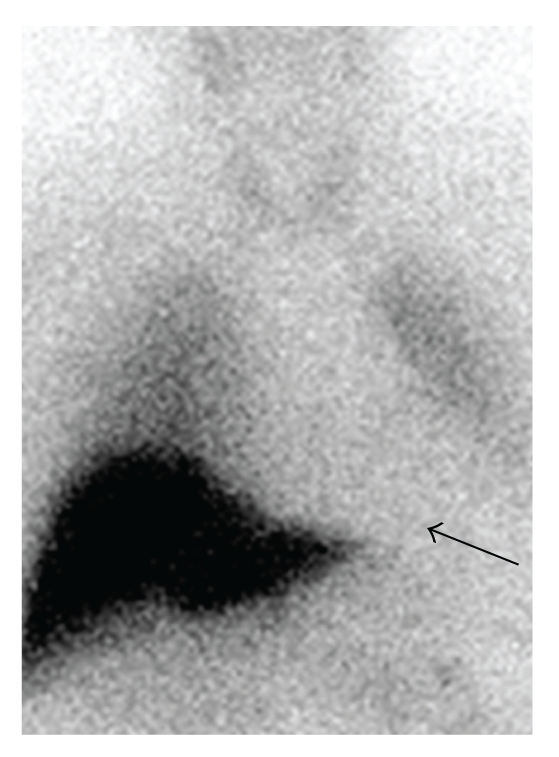
MIBG scintigraphy in akinetic rigid PD. MIBG scintigraphy in a 65-year old male patient with akinetic rigid type of PD, Hoehn and Yahr stage I. There is hardly any myocardial MIBG uptake (black arrow).

The myocardial MIBG scintigraphy measures the postganglionic sympathetic cardiac innervation (Figure 1). In the nineties of the last century it was observed that patients with Parkinson's disease (PD) disclose a significantly lower myocardial MIBG uptake than healthy controls (Figures 1–3, [9, 10]). This observation of a reduced myocardial MIBG uptake in PD was reproduced in numerous studies (Table 1). The reduced myocardial MIBG uptake reflects the sympathetic myocardial degeneration in PD and results from a Lewy body type-degeneration of the cardiac plexus [11–13]. MIBG scintigraphy is measured 15 minutes (early registration) and four hours (late registration) after intravenous MIBG application. The early registration mainly correlates with the influx of MIBG into extraneural spaces in the myocardial tissue rather than into neural components. The late registration displays the neuronal uptake of MIBG more explicitly and correlates with the functional status of sympathetic nerve terminals [14–16]. Therefore the late registration is more relevant in clinical practice [17]. All data in the following refer to the late registration. The myocardial MIBG uptake is quantified by the relation between *MIBG uptake in the heart* (more exactly: myocardium) versus *MIBG uptake in the mediastinum*. The heart-to-mediastinum ratio (H/M ratio) is calculated as the quotient *[counts per pixel in the myocardium]/[counts per pixel in the upper mediastium]* [17, 18]. PD patients show a significantly lower H/M ratio than healthy controls [9–13]. For this reason, MIBG scintigraphy may be helpful in the diagnosis of PD. Most studies did not find any significant correlation between myocardial MIBG uptake versus gender [19], age, disease duration or Hoehn and Yahr stage in PD [18, 20–24].

## 3. Factors Interfering with Myocardial MIBG Scintigraphy

Coronary heart disease represents an exclusion criterion for myocardial MIBG scintigraphy, since a myocardial hypoperfusion due to coronary heart disease may influence myocardial MIBG scintigraphy. 99mTc-Metoxy-Isopropyl-Isonitril (MIBI) scintigraphy can detect a local myocardial hypoperfusion [25]. Therefore MIBI scintigraphy, prior to MIBG scintigraphy, is meaningful to exclude a myocardial hypoperfusion. Primary or secondary cardiomyopathies of each type are further exclusion criteria for MIBG scintigraphy [26–30]. In addition, several drugs may influence myocardial MIBG uptake and should be paused before the MIBG study: for instance, tricyclic and tetracyclic antidepressants as well as reserpine block myocardial MIBG uptake. Calcium antagonists increase or prolong the myocardial uptake of MIBG [14, 31, 32]. A detailed list of “forbidden” drugs was published by Solanki et al. [32].

## 4. Sensitivity and Specificity of MIBG Scintigraphy

PD is diagnosed clinically. The UK Brain Bank criteria [33] represent the most frequently applied clinical criteria for diagnosis of PD. The UK Brain Bank criteria were defined by clinical intravitam symptoms of these patients, in whom PD diagnosis was confirmed neuropathologically. The specificity of these criteria amounts to 93% [34]. This means that—despite a conscientious clinical examination—7% of clinically diagnosed PD patients have—in reality—no PD. This fact requires the use of nuclear medicine methods in diagnostically unclear patients. Most studies found a high sensitivity of MIBG scintigraphy in the Hoehn and Yahr stage I of PD. However, a previous study [35] claimed a broad overlap between healthy controls and PD patients at Hoehn and Yahr stage I indicating a rather low sensitivity in the initial stage of PD; the published data of this study do not allow the calculation of sensitivity or specificity of MIBG scintigraphy in this study. To sum up one may say that a reduced myocardial MIBG uptake supports support the clinical diagnosis of PD even in the initial stage of PD. In the more advanced Hoehn and Yahr stages II–V, MIBG scintigraphy reveals a high sensitivity for PD (Table 1). The followingsections address the specificity of MIBG scintigraphyconcerning PD versus other extrapyramidal diseases.

### 4.1. Lewy Body Dementia

PD and Lewy body dementia (LBD) share a common pathology with a Lewy body type-degeneration of the nervous system. The anatomical distribution of this Lewy body type-degeneration—in PD primarily in the basal ganglia, in LBD primarily in cortical areas—decides whether the patient shows clinical symptoms of PD or LBD. Both diseases, PD and LBD, are characterized by a remarkable Lewy body type-degeneration of postganglionic myocardial sympathetic neurons. Therefore MIBG scintigraphy discloses a pathologically reduced myocardial MIBG uptake in both PD and LBD patients [13, 39]. Myocardial MIBG uptake is slightly more reduced in LBD than in PD patients [23, 40]. But due to the broad overlap between LBD and PD, myocardial MIBG scintigraphy cannot differentiate between both diseases [13, 23, 35, 39, 40].

### 4.2. Hereditary Parkinsonism

There exist only small data to MIBG scintigraphy in hereditary Parkinsonism (HP): Quattrone et al. [41] reported a normal myocardial MIBG uptake in all ten healthy subjects, eight of 14 HP patients, but none of 15 PD patients. Orimo et al. [42] found a normal MIBG scintigraphy in two HP patients with a homozygous PARK2 mutation. These studies suggest that MIBG scintigraphy is normal or less impaired in HP. Patients, who fulfil the UK Brain Bank criteria for PD but disclose a normal MIBG scintigraphy, might have HP. The family history may be inconspicuous, since autosomal recessive forms of HP occur.

### 4.3. Multiple System Atrophy

In multiple system atrophy (MSA), the autonomic nervous system is mainly affected in its preganglionic structures, whereas postganglionic involvement of the autonomic nervous system predominates in PD [43]. Following this pathoanatomical differences, it might be expected that myocardial MIBG uptake is reduced in PD and normal in MSA. Seen from this angle, MIBG scintigraphy might be suitable to distinguish between PD and MSA. Indeed, most MSA patients show a normal myocardial MIBG uptake [14, 24, 35–37, 44, 45]. The specificity of MIBG scintigraphy concerning PD against MSA varies between 0.70 and 0.95 ([35]: 0.79, [44]: 0.70, [14]: 0.95, [37]: 0.86, [24]: 0.78, [36]: 0.80).

### 4.4. Corticobasal Degeneration

Orimo et al. [46] reported a normal myocardial MIBG scintigraphy in all eight patients with corticobasal degeneration (CBD), which was not significantly different from that of healthy controls. Taki et al. [24] found a normal myocardial MIBG uptake in two CBD patients. These small case series might suggest that MIBG scintigraphy differentiates between PD and CBD, but this difference has to be confirmed in larger patient groups.

### 4.5. Progressive Supranuclear Palsy

Small case series disclosed contradictory findings: Nagayama et al. [35] observed a reduced myocardial MIBG uptake in six of seven patients with progressive supranuclear palsy (PSP), whereas Taki et al. [24] and Yoshita [36] described a reduced myocardial MIBG uptake only in one of six and three of 14 patients, respectively. Similar to CBD, studies with larger case numbers are necessary to clarify the diagnostic value of MIBG scintigraphy in PSP.

### 4.6. Essential Tremor

Patients with essential tremor (ET) reveal a normal myocardial MIBG uptake, as reported by Lee et al. [47] in 22 ET patients and by Orimo et al. [37] in five ET patients. MIBG scintigraphy clearly distinguishes patients with ET from patients with PD, even from patients with early PD or tremor-dominant PD which is sometimes very similar to ET. 

## 5. Correlation of MIBG Scintigraphy with Clinical Parameters

### 5.1. Autonomous Functions

Since myocardial MIBG scintigraphy measures the sympathetic myocardial function, it is of interest whether myocardial MIBG uptake correlates with other autonomous functions. The autonomous nervous system can be investigated by means of several clinical tests such as orthostatic reaction, deep breathing, or Valsalva manoeuvre. During these clinical tests, the blood pressure (regulated mainly by the sympathetic nervous system), the heart rate, and variability of heart rate (regulated mainly by the parasympathetic nervous system) are measured [48]. There are contradicting findings concerning the relation between myocardial MIBG uptake and functional integrity of the autonomous nervous system: Oka et al. [49], Spiegel et al. [38], and Shibata et al. [22] observed a significant correlation between myocardial MIBG uptake and both sympathetic and parasympathetic function. In contrast to that, Matsui et al. [21], Haensch et al. [50], and Doi et al. [51] did not find any significant correlation between myocardial MIBG uptake and sympathetic or parasympathetic function. These inconsistent findings may be based on the different methods to test the autonomous functions.

### 5.2. Motor Phenotype of PD

Myocardial MIBG uptake correlates with motor symptoms in PD; patients with tremor dominant PD reveal a significantly higher MIBG uptake (Figure 2) than patients with the hypokinetic rigid type of PD at each Hoehn and Yahr stage (Figure 3; [38, 52]). Myocardial MIBG uptake correlates significantly with the severity of hypokinesia but not with the extent of resting or postural tremor [19, 20, 38].

## 6. Pathophysiological Impact of MIBG Scintigraphy

PD is characterized by a cerebral as well as extracranial Lewy body type-degeneration. The cerebral—predominantly nigrostriatal dopaminergic—Lewy body degenerations is usually visualized by cerebral SPECT of the presynaptic dopamine transporters (DAT). As mentioned above, MIBG scintigraphy quantifies exactly the extent of extracranial myocardial sympathetic Lewy body degeneration. It is unclear whether cerebral nigrostriatal dopaminergic and extracranial sympathetic Lewy body degeneration occur independently from each other or not. “Extreme” cases might be possible in whom only the cerebral nigrostriatal system but not the extracranial sympathetic system degenerates or vice versa. The intraindividual comparison between cerebral nigrostriatal DAT SPECT and myocardial MIBG scintigraphy may clarify this aspect: Spiegel et al. [53] and Spiegel et al. [18] stated a significant correlation between cerebral nigrostriatal dopaminergic and extracranial myocardial sympathetic degeneration at each Hoehn and Yahr stage. No such correlation was found in healthy controls. These findings suggest that cerebral and extracranial Lewy body degenerations develop in a closely coupled manner in PD.

## 7. Conclusions

Myocardial MIBG scintigraphy helps to differentiate between PD and other parkinsonian syndromes in clinically difficult cases. Furthermore, the myocardial MIBG scintigraphy allows insights into the pathophysiology of PD; myocardial MIBG uptake correlates significantly with the motor phenotype and with the nigrostriatal function (measured by DAT SPECT). These facts suggest that cerebral nigrostriatal dopaminergic degeneration and myocardial sympathetic degeneration are closely coupled in PD. 

## Figures and Tables

**Table 1:** Sensitivity and specificity of MIBG scintigraphy in Parkinson's disease. N: total number of investigated patients in the cited study; APS: atypical Parkinsonian syndromes; Mean H & Y: arithmetical mean of Hoehn and Yahr stage of the PD patients. The strongly varying specificity may be caused by the variable distribution of the diagnoses of the patients with atypical parkinsonian syndromes. The lower norm value was calculated as mean −2.5 standard deviations (if not declared otherwise in the original papers). All data belong to the delayed imaging four hours after intravenous MIBG application.

Study	N	Patients with PD/Hoehn and Yahr stage	Patients with APS	Sensitivity	Specificity
Yoshita [36]	54	*n* = 25/mean H & Y = 2.1	*n* = 29	1.00	0.79
Orimo et al. [37]	68	*n* = 45/mean H & Y = 3.0	*n* = 23	0.80	0.87
Taki et al. [24]	70	*n* = 41/mean H & Y = 1.9	*n* = 29	0.90	0.76
Braune [14]	291	*n* = 246/H & Y ≥ 2	*n* = 45	0.90	0.94
Spiegel et al. [38]	102	*n* = 102/mean H & Y = 1,7	—	0.96	—
Sewada et al. [17]	400	*n* = 267/mean H & Y = 3.2	*n* = 133	0.84	0.89
